# Bis(pyridine-2-carbaldehyde thio­semi­carbazone)zinc(II) dinitrate dihydrate

**DOI:** 10.1107/S1600536810038614

**Published:** 2010-09-30

**Authors:** Lin Cheng, Li-Min Zhang, Jian-Quan Wang

**Affiliations:** a Department of Chemistry and Chemical Engineering, Southeast University, Nanjing 211189, People’s Republic of China

## Abstract

The asymmetric unit of the title compound, [Zn(C_7_H_8_N_4_S)_2_](NO_3_)_2_·2H_2_O, contains two Zn(pht)_2_ cations (pht is pyridine-2-carbaldehyde thio­semicarbazone), four nitrate anions and four water mol­ecules. In the cations, each Zn^II^ ion adopts a distorted octa­hedral coordination geometry, being chelated by two tridentate pht ligands. In the crystal, the cations, anions and water mol­ecules are connected *via* O—H⋯O and N—H⋯O hydrogen bonds into a three-dimensional network.

## Related literature

For related structures, see: Antholine *et al.* (1977 ▶); Ainscough *et al.* (1998 ▶).
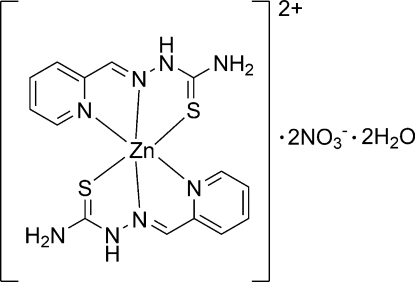


## Experimental

### 

#### Crystal data

[Zn(C_7_H_8_N_4_S)_2_](NO_3_)_2_·2H_2_O
*M* *_r_* = 585.89
Monoclinic, 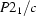

*a* = 21.4623 (14) Å
*b* = 16.6324 (12) Å
*c* = 13.2764 (10) Å
β = 102.876 (2)°
*V* = 4620.1 (6) Å^3^
*Z* = 8
Mo *K*α radiation
μ = 1.31 mm^−1^
*T* = 120 K
0.25 × 0.22 × 0.20 mm

#### Data collection

Bruker SMART APEX CCD diffractometer
Absorption correction: multi-scan (*SADABS*; Sheldrick, 2008*a* ▶) *T*_min_ = 0.736, *T*_max_ = 0.780
22904 measured reflections
8732 independent reflections
5113 reflections with *I* > 2σ(*I*)
*R*_int_ = 0.077

#### Refinement

*R*[*F* ^2^ > 2σ(*F* ^2^)] = 0.057
*wR*(*F* ^2^) = 0.123
*S* = 1.02
8732 reflections
631 parameters
H-atom parameters constrained
Δρ_max_ = 0.81 e Å^−3^
Δρ_min_ = −0.43 e Å^−3^

### 

Data collection: *SMART* (Bruker, 2000 ▶); cell refinement: *SAINT* (Bruker, 2000 ▶); data reduction: *SAINT*; program(s) used to solve structure: *SHELXTL* (Sheldrick, 2008*b* ▶); program(s) used to refine structure: *SHELXL97* (Sheldrick, 2008*b* ▶); molecular graphics: *ORTEPIII* (Burnett & Johnson, 1996 ▶) and *PLATON* (Spek, 2009 ▶); software used to prepare material for publication: *SHELXL97*.

## Supplementary Material

Crystal structure: contains datablocks I, global. DOI: 10.1107/S1600536810038614/bt5358sup1.cif

Structure factors: contains datablocks I. DOI: 10.1107/S1600536810038614/bt5358Isup2.hkl

Additional supplementary materials:  crystallographic information; 3D view; checkCIF report

## Figures and Tables

**Table 1:** Hydrogen-bond geometry (Å, °)

*D*—H⋯*A*	*D*—H	H⋯*A*	*D*⋯*A*	*D*—H⋯*A*
N3—H3*B*⋯O7	0.86	2.19	2.966 (6)	150
N4—H4*B*⋯O7	0.86	2.17	2.957 (6)	153
N4—H4*C*⋯O3*W*	0.86	2.03	2.840 (5)	157
N7—H7*A*⋯O1	0.86	1.96	2.813 (6)	172
N8—H8*A*⋯O2	0.86	2.08	2.906 (6)	160
N8—H8*B*⋯O6^i^	0.86	2.04	2.870 (6)	163
N11—H11*B*⋯O10	0.86	1.87	2.710 (5)	167
N12—H12*B*⋯O11	0.86	2.02	2.876 (6)	173
N12—H12*C*⋯O8^ii^	0.86	2.27	3.114 (6)	168
N15—H15*A*⋯O4*W*^iii^	0.86	1.87	2.733 (5)	178
N16—H16*A*⋯O6^iii^	0.86	2.11	2.909 (6)	155
N16—H16*B*⋯O4^iv^	0.86	2.02	2.873 (5)	173
O1*W*—H1*WA*⋯O4^v^	0.85	2.32	3.042 (5)	144
O1*W*—H1*WB*⋯O3	0.85	2.39	3.041 (6)	134
O2*W*—H2*WA*⋯O5^iv^	0.85	2.33	2.927 (6)	128
O2*W*—H2*WB*⋯O3	0.85	2.47	3.141 (6)	136
O3*W*—H3*WA*⋯O10^vi^	0.85	2.22	2.800 (5)	126
O3*W*—H3*WB*⋯O9^vii^	0.85	2.24	2.978 (6)	145
O4*W*—H4*WA*⋯O2*W*	0.85	2.09	2.746 (6)	133
O4*W*—H4*WB*⋯O6	0.85	2.55	2.958 (5)	111

Symmetry codes: (i) 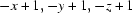; (ii) 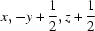; (iii) 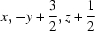; (iv) 
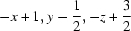
; (v) 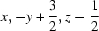; (vi) 
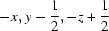
; (vii) 
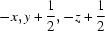
.

**Fig. 1.**
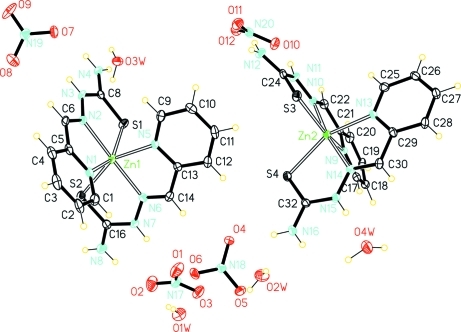
View of the asymmetric unit of the title compoundcompound with the atom-labelling scheme. Displacement ellipsoids are drawn at the 30% probability level. H atoms are represented as small spheres of arbitrary radii.

## supplementary crystallographic information

### Comment

The examination of the antitumor properties of N-heterocyclic carboxaldehyde thiosemicarbazones has been extended to the consideration of some of their first-row transition metal complexes (Antholine *et al.* 1977, Ainscough *et al.* 1998). Meanwhile, zinc plays an important role in various biological systems and is a vital component an essential cofactor, critical for numerous cellular processes and may be a major regulatory ion in the metabolism of cells. Herein, we report a Zn^II^ complex, Zn(pht)_2_.2NO_3_.2H_2_O (pht= 2-(pyridine-2-carbaldehyde)hydrazinecarbothioamide).

The asymmetric unit of the title compound, contains two Zn(pht)_2_ cations (pht = 2-(pyridine-2-carbaldehyde)hydrazinecarbothioamide), which have the similar structure, four nitrates and four free water molecules. In the Zn(pht)_2_ cations, each Zn^II^ ions adopts a distorted octahedral coordination geometry, being chelated by two tridentate pht ligands. In a pht, all the atoms are approximatively coplanar, and the distances of the C=N bond are 1.269 (6)–1.283 (6) Å, which are shorter than those of C—N bond (1.303 (7)–1.369 (6) Å), being considered to have full double-bond character. In packing, All the Zn(pht)_2_ cations, nitrates and water molecules are linked each other *via* O—H···O and N—H···O hydrogen bonds into a three-dimensional supramolecular network.

### Experimental

A mixture of pht (0.036 g, 0.2 mmol), Zn(NO_3_)_2_.6H_2_O (0.029 g, 0.1 mmol), and water (8 ml) were heated ina 15 ml Teflon-lined vessel at 120 ° for 3 days, followed by slow cooling (5 ° h^-1^) to room temperature. After filtration, colorless block crystals were collected and dried in air (0.018 g, yield *ca* 30.7% based on pht).

### Refinement

All H atoms attached to C and N atoms were fixed geometrically and treated as riding with C—H = 0.93 Å and N—H = 0.86 Å with *U*_iso_(H) = 1.2 *U*_eq_(C or N). H atoms of water molecules were located in difference Fourier maps and included in the subsequent refinement with O—H= 0.85Å with *U*_iso_(H) = 1.2 *U*_eq_(O).

### Crystal data

**Table:**

[Zn(C_7_H_8_N_4_S)_2_](NO_3_)_2_·2H_2_O	*F*(000) = 2400
*M**_r_* = 585.89	*D*_x_ = 1.685 Mg m^−^^3^
Monoclinic, *P*2_1_/*c*	Mo *K*α radiation, λ = 0.71073 Å
Hall symbol: -P 2ybc	Cell parameters from 785 reflections
*a* = 21.4623 (14) Å	θ = 2.4–28.0°
*b* = 16.6324 (12) Å	µ = 1.31 mm^−^^1^
*c* = 13.2764 (10) Å	*T* = 120 K
β = 102.876 (2)°	Block, colorless
*V* = 4620.1 (6) Å^3^	0.25 × 0.22 × 0.20 mm
*Z* = 8	

### Data collection

**Table:**

Bruker SMART APEX CCD diffractometer	8732 independent reflections
Radiation source: fine-focus sealed tube	5113 reflections with *I* > 2σ(*I*)
graphite	*R*_int_ = 0.077
φ and ω scan	θ_max_ = 26.0°, θ_min_ = 1.6°
Absorption correction: multi-scan (*SADABS*; Sheldrick, 2008a)	*h* = −25→21
*T*_min_ = 0.736, *T*_max_ = 0.780	*k* = −20→12
22904 measured reflections	*l* = −16→16

### Refinement

**Table:**

Refinement on *F*^2^	Primary atom site location: structure-invariant direct methods
Least-squares matrix: full	Secondary atom site location: difference Fourier map
*R*[*F*^2^ > 2σ(*F*^2^)] = 0.057	Hydrogen site location: inferred from neighbouring sites
*wR*(*F*^2^) = 0.123	H-atom parameters constrained
*S* = 1.02	*w* = 1/[σ^2^(*F*_o_^2^) + (0.0354*P*)^2^] where *P* = (*F*_o_^2^ + 2*F*_c_^2^)/3
8732 reflections	(Δ/σ)_max_ = 0.029
631 parameters	Δρ_max_ = 0.81 e Å^−^^3^
0 restraints	Δρ_min_ = −0.43 e Å^−^^3^

### Special details

**Table:**

Geometry. All e.s.d.'s (except the e.s.d. in the dihedral angle between two l.s. planes) are estimated using the full covariance matrix. The cell e.s.d.'s are taken into account individually in the estimation of e.s.d.'s in distances, angles and torsion angles; correlations between e.s.d.'s in cell parameters are only used when they are defined by crystal symmetry. An approximate (isotropic) treatment of cell e.s.d.'s is used for estimating e.s.d.'s involving l.s. planes.
Refinement. Refinement of *F*^2^ against ALL reflections. The weighted *R*-factor *wR* and goodness of fit *S* are based on *F*^2^, conventional *R*-factors *R* are based on *F*, with *F* set to zero for negative *F*^2^. The threshold expression of *F*^2^ > σ(*F*^2^) is used only for calculating *R*-factors(gt) *etc*. and is not relevant to the choice of reflections for refinement. *R*-factors based on *F*^2^ are statistically about twice as large as those based on *F*, and *R*- factors based on ALL data will be even larger.

### Fractional atomic coordinates and isotropic or equivalent isotropic displacement parameters (Å^2^)

**Table:**

	*x*	*y*	*z*	*U*_iso_*/*U*_eq_	
Zn1	0.25244 (3)	0.23919 (3)	0.57658 (4)	0.02381 (17)	
Zn2	0.22452 (3)	0.75501 (3)	0.82766 (4)	0.02208 (17)	
S1	0.18156 (7)	0.30117 (8)	0.42437 (10)	0.0301 (4)	
S2	0.32904 (7)	0.17217 (8)	0.48824 (11)	0.0330 (4)	
S3	0.14317 (7)	0.65650 (8)	0.85347 (10)	0.0280 (3)	
S4	0.30520 (7)	0.65526 (8)	0.80976 (10)	0.0280 (4)	
C1	0.3251 (3)	0.1529 (3)	0.7846 (4)	0.0339 (15)	
H1A	0.3582	0.1887	0.7831	0.041*	
C2	0.3318 (3)	0.0998 (3)	0.8668 (4)	0.0378 (16)	
H2A	0.3684	0.1004	0.9195	0.045*	
C3	0.2829 (3)	0.0460 (3)	0.8692 (4)	0.0386 (16)	
H3A	0.2863	0.0094	0.9231	0.046*	
C4	0.2294 (3)	0.0477 (3)	0.7902 (4)	0.0332 (15)	
H4A	0.1959	0.0123	0.7900	0.040*	
C5	0.2258 (3)	0.1028 (3)	0.7106 (4)	0.0266 (13)	
C6	0.1702 (3)	0.1088 (3)	0.6264 (4)	0.0277 (13)	
H6A	0.1362	0.0731	0.6202	0.033*	
C8	0.1178 (2)	0.2397 (3)	0.4168 (4)	0.0223 (12)	
C9	0.1696 (3)	0.3390 (3)	0.7026 (4)	0.0270 (13)	
H9A	0.1368	0.3033	0.6752	0.032*	
C10	0.1577 (3)	0.4020 (3)	0.7660 (4)	0.0285 (14)	
H10A	0.1173	0.4086	0.7797	0.034*	
C11	0.2065 (3)	0.4540 (3)	0.8076 (4)	0.0330 (15)	
H11A	0.1996	0.4961	0.8499	0.040*	
C12	0.2655 (3)	0.4431 (3)	0.7859 (4)	0.0298 (14)	
H12A	0.2992	0.4773	0.8137	0.036*	
C13	0.2735 (3)	0.3807 (3)	0.7222 (3)	0.0200 (12)	
C14	0.3341 (3)	0.3668 (3)	0.6934 (4)	0.0256 (13)	
H14A	0.3701	0.3973	0.7214	0.031*	
C16	0.3920 (3)	0.2337 (3)	0.5309 (4)	0.0288 (13)	
C17	0.3197 (3)	0.8776 (3)	0.7546 (4)	0.0286 (14)	
H17A	0.3479	0.8639	0.8161	0.034*	
C18	0.3399 (3)	0.9322 (3)	0.6897 (4)	0.0300 (14)	
H18A	0.3812	0.9530	0.7059	0.036*	
C19	0.2978 (3)	0.9545 (3)	0.6017 (4)	0.0334 (15)	
H19A	0.3097	0.9922	0.5577	0.040*	
C20	0.2369 (3)	0.9208 (3)	0.5776 (4)	0.0291 (14)	
H20A	0.2073	0.9359	0.5182	0.035*	
C21	0.2214 (2)	0.8643 (3)	0.6445 (4)	0.0207 (12)	
C22	0.1609 (3)	0.8202 (3)	0.6203 (4)	0.0256 (13)	
H22A	0.1292	0.8320	0.5621	0.031*	
C24	0.0965 (3)	0.6578 (3)	0.7329 (4)	0.0232 (13)	
C25	0.1337 (3)	0.8917 (3)	0.8776 (4)	0.0278 (13)	
H25A	0.1075	0.8782	0.8141	0.033*	
C26	0.1147 (3)	0.9538 (3)	0.9340 (4)	0.0285 (14)	
H26A	0.0771	0.9819	0.9077	0.034*	
C27	0.1520 (3)	0.9729 (3)	1.0289 (4)	0.0320 (14)	
H27A	0.1398	1.0135	1.0686	0.038*	
C28	0.2086 (3)	0.9305 (3)	1.0648 (4)	0.0320 (14)	
H28A	0.2350	0.9427	1.1286	0.038*	
C29	0.2246 (3)	0.8702 (3)	1.0038 (4)	0.0235 (13)	
C30	0.2841 (3)	0.8244 (3)	1.0344 (4)	0.0258 (13)	
H30A	0.3132	0.8343	1.0963	0.031*	
C32	0.3575 (3)	0.6699 (3)	0.9239 (4)	0.0242 (13)	
N1	0.2738 (2)	0.1546 (2)	0.7083 (3)	0.0270 (11)	
N2	0.1698 (2)	0.1650 (2)	0.5611 (3)	0.0234 (10)	
N3	0.1180 (2)	0.1775 (2)	0.4824 (3)	0.0273 (11)	
H3B	0.0855	0.1460	0.4745	0.033*	
N4	0.06559 (19)	0.2474 (2)	0.3439 (3)	0.0275 (11)	
H4B	0.0346	0.2140	0.3405	0.033*	
H4C	0.0624	0.2858	0.2997	0.033*	
N5	0.2263 (2)	0.3291 (2)	0.6810 (3)	0.0225 (10)	
N6	0.3358 (2)	0.3111 (2)	0.6284 (3)	0.0243 (11)	
N7	0.3911 (2)	0.2952 (2)	0.5973 (3)	0.0287 (11)	
H7A	0.4247	0.3239	0.6195	0.034*	
N8	0.4457 (2)	0.2266 (3)	0.4975 (4)	0.0427 (13)	
H8A	0.4766	0.2598	0.5183	0.051*	
H8B	0.4495	0.1888	0.4552	0.051*	
N9	0.2622 (2)	0.8438 (2)	0.7334 (3)	0.0231 (10)	
N10	0.15389 (19)	0.7643 (2)	0.6836 (3)	0.0195 (10)	
N11	0.1020 (2)	0.7152 (2)	0.6629 (3)	0.0244 (11)	
H11B	0.0732	0.7204	0.6067	0.029*	
N12	0.0521 (2)	0.6028 (2)	0.7020 (3)	0.0316 (12)	
H12B	0.0286	0.6047	0.6404	0.038*	
H12C	0.0466	0.5650	0.7433	0.038*	
N13	0.1877 (2)	0.8507 (2)	0.9102 (3)	0.0229 (10)	
N14	0.2938 (2)	0.7703 (2)	0.9722 (3)	0.0212 (10)	
N15	0.3479 (2)	0.7262 (2)	0.9943 (3)	0.0256 (11)	
H15A	0.3757	0.7333	1.0511	0.031*	
N16	0.4098 (2)	0.6278 (2)	0.9496 (3)	0.0328 (12)	
H16A	0.4360	0.6362	1.0079	0.039*	
H16B	0.4182	0.5916	0.9083	0.039*	
N17	0.5277 (3)	0.4141 (3)	0.5916 (4)	0.0421 (13)	
N18	0.5183 (2)	0.9676 (3)	0.6417 (3)	0.0291 (11)	
N19	−0.0127 (3)	0.0485 (3)	0.3698 (4)	0.0358 (12)	
N20	−0.0031 (2)	0.6531 (3)	0.4316 (4)	0.0358 (12)	
O1	0.4955 (2)	0.4005 (3)	0.6560 (3)	0.0628 (14)	
O2	0.5329 (2)	0.3626 (3)	0.5256 (4)	0.0677 (15)	
O3	0.5557 (2)	0.4799 (3)	0.5909 (3)	0.0544 (13)	
O4	0.57090 (17)	0.9985 (2)	0.6837 (3)	0.0329 (10)	
O5	0.46998 (18)	1.0110 (2)	0.6105 (3)	0.0406 (11)	
O6	0.51370 (17)	0.8926 (2)	0.6304 (3)	0.0335 (10)	
O7	−0.01433 (19)	0.1186 (2)	0.4029 (3)	0.0448 (11)	
O8	0.0395 (2)	0.0142 (3)	0.3794 (3)	0.0516 (12)	
O9	−0.0623 (2)	0.0145 (3)	0.3268 (3)	0.0648 (15)	
O10	0.02421 (18)	0.7177 (2)	0.4717 (3)	0.0395 (11)	
O11	−0.01805 (19)	0.6012 (2)	0.4904 (3)	0.0430 (11)	
O12	−0.01414 (18)	0.6431 (2)	0.3370 (3)	0.0441 (11)	
O1W	0.62343 (19)	0.4712 (3)	0.4129 (3)	0.0608 (13)	
H1WA	0.6112	0.4562	0.3504	0.073*	
H1WB	0.6057	0.4444	0.4533	0.073*	
O2W	0.52013 (19)	0.6250 (3)	0.7187 (3)	0.0588 (13)	
H2WA	0.5064	0.6175	0.7733	0.071*	
H2WB	0.5496	0.5995	0.6990	0.071*	
O3W	0.04209 (17)	0.3374 (2)	0.1575 (3)	0.0400 (10)	
H3WA	0.0152	0.3325	0.1001	0.048*	
H3WB	0.0337	0.3847	0.1746	0.048*	
O4W	0.43806 (18)	0.7533 (2)	0.6735 (3)	0.0542 (12)	
H4WA	0.4679	0.7266	0.6564	0.065*	
H4WB	0.4545	0.7897	0.7161	0.065*	

### Atomic displacement parameters (Å^2^)

**Table:**

	*U*^11^	*U*^22^	*U*^33^	*U*^12^	*U*^13^	*U*^23^
Zn1	0.0256 (4)	0.0197 (3)	0.0261 (3)	−0.0050 (3)	0.0059 (3)	−0.0023 (3)
Zn2	0.0243 (4)	0.0183 (3)	0.0222 (3)	0.0012 (3)	0.0023 (3)	−0.0007 (3)
S1	0.0311 (10)	0.0296 (8)	0.0283 (7)	−0.0100 (7)	0.0039 (7)	0.0025 (6)
S2	0.0338 (10)	0.0279 (8)	0.0388 (8)	−0.0050 (7)	0.0116 (7)	−0.0111 (6)
S3	0.0340 (10)	0.0228 (7)	0.0260 (7)	−0.0015 (7)	0.0038 (6)	0.0007 (6)
S4	0.0302 (9)	0.0229 (7)	0.0279 (7)	0.0048 (7)	0.0005 (6)	−0.0041 (6)
C1	0.040 (4)	0.024 (3)	0.034 (3)	−0.002 (3)	0.001 (3)	−0.003 (3)
C2	0.059 (5)	0.025 (3)	0.028 (3)	0.016 (3)	0.006 (3)	−0.005 (3)
C3	0.065 (5)	0.021 (3)	0.036 (3)	0.007 (3)	0.025 (3)	0.004 (3)
C4	0.045 (4)	0.021 (3)	0.038 (3)	0.008 (3)	0.019 (3)	0.003 (3)
C5	0.035 (4)	0.017 (3)	0.034 (3)	0.004 (3)	0.019 (3)	0.001 (2)
C6	0.029 (4)	0.015 (3)	0.043 (3)	−0.005 (3)	0.015 (3)	−0.001 (3)
C8	0.023 (3)	0.020 (3)	0.025 (3)	0.002 (3)	0.008 (2)	−0.006 (2)
C9	0.038 (4)	0.018 (3)	0.025 (3)	−0.004 (3)	0.008 (3)	0.004 (2)
C10	0.032 (4)	0.031 (3)	0.026 (3)	0.010 (3)	0.012 (3)	0.012 (2)
C11	0.055 (5)	0.018 (3)	0.025 (3)	0.007 (3)	0.005 (3)	0.000 (2)
C12	0.041 (4)	0.019 (3)	0.028 (3)	−0.002 (3)	0.004 (3)	−0.003 (2)
C13	0.023 (4)	0.019 (3)	0.017 (3)	0.002 (3)	0.004 (2)	0.003 (2)
C14	0.030 (4)	0.021 (3)	0.023 (3)	−0.005 (3)	0.001 (3)	0.002 (2)
C16	0.029 (4)	0.024 (3)	0.034 (3)	0.002 (3)	0.009 (3)	−0.001 (2)
C17	0.027 (4)	0.022 (3)	0.035 (3)	0.005 (3)	0.004 (3)	−0.005 (3)
C18	0.025 (4)	0.023 (3)	0.044 (3)	−0.003 (3)	0.012 (3)	−0.005 (3)
C19	0.051 (4)	0.021 (3)	0.034 (3)	−0.013 (3)	0.022 (3)	−0.006 (3)
C20	0.040 (4)	0.026 (3)	0.023 (3)	−0.005 (3)	0.013 (3)	0.000 (2)
C21	0.023 (3)	0.014 (3)	0.027 (3)	0.002 (2)	0.008 (2)	−0.003 (2)
C22	0.031 (4)	0.024 (3)	0.022 (3)	0.002 (3)	0.006 (3)	0.001 (2)
C24	0.030 (4)	0.016 (3)	0.027 (3)	0.002 (3)	0.012 (3)	−0.004 (2)
C25	0.025 (4)	0.026 (3)	0.033 (3)	−0.002 (3)	0.007 (3)	0.003 (3)
C26	0.024 (4)	0.024 (3)	0.039 (3)	0.006 (3)	0.011 (3)	0.002 (3)
C27	0.034 (4)	0.022 (3)	0.043 (3)	0.001 (3)	0.015 (3)	−0.009 (3)
C28	0.037 (4)	0.022 (3)	0.038 (3)	−0.005 (3)	0.010 (3)	−0.011 (3)
C29	0.028 (4)	0.017 (3)	0.028 (3)	0.002 (3)	0.012 (3)	0.001 (2)
C30	0.033 (4)	0.024 (3)	0.021 (3)	0.000 (3)	0.007 (3)	−0.005 (2)
C32	0.019 (3)	0.020 (3)	0.035 (3)	0.000 (3)	0.010 (3)	0.001 (2)
N1	0.030 (3)	0.019 (2)	0.034 (3)	0.003 (2)	0.011 (2)	0.000 (2)
N2	0.027 (3)	0.016 (2)	0.029 (2)	−0.002 (2)	0.009 (2)	0.0015 (19)
N3	0.025 (3)	0.020 (2)	0.036 (3)	−0.009 (2)	0.005 (2)	−0.003 (2)
N4	0.021 (3)	0.025 (3)	0.034 (2)	−0.004 (2)	0.001 (2)	0.001 (2)
N5	0.024 (3)	0.019 (2)	0.025 (2)	0.001 (2)	0.007 (2)	−0.0008 (19)
N6	0.026 (3)	0.021 (2)	0.028 (2)	0.001 (2)	0.011 (2)	0.003 (2)
N7	0.023 (3)	0.028 (3)	0.035 (3)	−0.005 (2)	0.005 (2)	−0.006 (2)
N8	0.032 (3)	0.042 (3)	0.057 (3)	−0.006 (3)	0.017 (3)	−0.016 (3)
N9	0.020 (3)	0.019 (2)	0.031 (2)	−0.002 (2)	0.007 (2)	−0.0047 (19)
N10	0.020 (3)	0.017 (2)	0.022 (2)	−0.001 (2)	0.0064 (19)	−0.0004 (18)
N11	0.022 (3)	0.026 (3)	0.023 (2)	−0.010 (2)	0.000 (2)	0.0038 (19)
N12	0.030 (3)	0.026 (3)	0.039 (3)	−0.011 (2)	0.007 (2)	0.002 (2)
N13	0.023 (3)	0.018 (2)	0.029 (2)	0.001 (2)	0.009 (2)	−0.0018 (19)
N14	0.017 (3)	0.021 (2)	0.025 (2)	0.002 (2)	0.0043 (19)	−0.0006 (19)
N15	0.028 (3)	0.023 (2)	0.021 (2)	0.001 (2)	−0.004 (2)	−0.0029 (18)
N16	0.031 (3)	0.028 (3)	0.036 (3)	0.007 (2)	0.000 (2)	−0.010 (2)
N17	0.041 (4)	0.041 (3)	0.042 (3)	−0.006 (3)	0.006 (3)	−0.006 (3)
N18	0.026 (3)	0.031 (3)	0.028 (3)	−0.003 (3)	0.002 (2)	0.004 (2)
N19	0.037 (4)	0.031 (3)	0.039 (3)	−0.005 (3)	0.009 (3)	0.007 (2)
N20	0.032 (3)	0.031 (3)	0.041 (3)	0.010 (3)	0.000 (3)	−0.001 (2)
O1	0.063 (3)	0.077 (4)	0.059 (3)	−0.036 (3)	0.038 (3)	−0.026 (3)
O2	0.081 (4)	0.059 (3)	0.075 (3)	−0.013 (3)	0.042 (3)	−0.022 (3)
O3	0.053 (3)	0.046 (3)	0.061 (3)	−0.020 (3)	0.006 (2)	−0.003 (2)
O4	0.023 (2)	0.030 (2)	0.043 (2)	−0.0044 (19)	0.0008 (19)	0.0046 (18)
O5	0.028 (3)	0.033 (2)	0.054 (3)	0.005 (2)	−0.005 (2)	0.001 (2)
O6	0.038 (3)	0.019 (2)	0.045 (2)	−0.0062 (19)	0.012 (2)	−0.0087 (18)
O7	0.051 (3)	0.021 (2)	0.072 (3)	−0.004 (2)	0.036 (2)	−0.008 (2)
O8	0.049 (3)	0.047 (3)	0.058 (3)	0.019 (3)	0.011 (2)	0.006 (2)
O9	0.065 (3)	0.059 (3)	0.058 (3)	−0.045 (3)	−0.014 (3)	0.015 (2)
O10	0.047 (3)	0.027 (2)	0.037 (2)	−0.004 (2)	−0.007 (2)	0.0000 (18)
O11	0.046 (3)	0.037 (2)	0.046 (3)	−0.008 (2)	0.010 (2)	0.007 (2)
O12	0.046 (3)	0.046 (3)	0.033 (2)	0.008 (2)	−0.006 (2)	−0.005 (2)
O1W	0.066 (3)	0.076 (3)	0.040 (3)	−0.008 (3)	0.010 (2)	0.004 (2)
O2W	0.048 (3)	0.070 (3)	0.061 (3)	0.002 (3)	0.018 (2)	0.005 (2)
O3W	0.046 (3)	0.034 (2)	0.040 (2)	−0.001 (2)	0.012 (2)	−0.0074 (19)
O4W	0.046 (3)	0.053 (3)	0.055 (3)	−0.010 (2)	−0.008 (2)	0.004 (2)

### Geometric parameters (Å, °)

**Table:**

Zn1—N6	2.133 (4)	C21—C22	1.463 (7)
Zn1—N2	2.133 (4)	C22—N10	1.283 (6)
Zn1—N5	2.196 (4)	C22—H22A	0.9300
Zn1—N1	2.211 (4)	C24—N12	1.319 (6)
Zn1—S1	2.4678 (15)	C24—N11	1.356 (6)
Zn1—S2	2.4860 (16)	C25—N13	1.331 (6)
Zn2—N10	2.165 (4)	C25—C26	1.389 (7)
Zn2—N14	2.165 (4)	C25—H25A	0.9300
Zn2—N13	2.178 (4)	C26—C27	1.372 (7)
Zn2—N9	2.203 (4)	C26—H26A	0.9300
Zn2—S4	2.4475 (15)	C27—C28	1.393 (7)
Zn2—S3	2.4733 (15)	C27—H27A	0.9300
S1—C8	1.692 (5)	C28—C29	1.380 (7)
S2—C16	1.689 (6)	C28—H28A	0.9300
S3—C24	1.688 (5)	C29—N13	1.356 (6)
S4—C32	1.690 (5)	C29—C30	1.464 (7)
C1—N1	1.319 (6)	C30—N14	1.270 (6)
C1—C2	1.386 (7)	C30—H30A	0.9300
C1—H1A	0.9300	C32—N16	1.304 (6)
C2—C3	1.385 (7)	C32—N15	1.369 (6)
C2—H2A	0.9300	N2—N3	1.361 (5)
C3—C4	1.372 (7)	N3—H3B	0.8600
C3—H3A	0.9300	N4—H4B	0.8600
C4—C5	1.388 (7)	N4—H4C	0.8600
C4—H4A	0.9300	N6—N7	1.367 (5)
C5—N1	1.349 (6)	N7—H7A	0.8600
C5—C6	1.445 (7)	N8—H8A	0.8600
C6—N2	1.273 (6)	N8—H8B	0.8600
C6—H6A	0.9300	N10—N11	1.359 (5)
C8—N4	1.314 (6)	N11—H11B	0.8600
C8—N3	1.353 (6)	N12—H12B	0.8600
C9—N5	1.322 (6)	N12—H12C	0.8600
C9—C10	1.404 (7)	N14—N15	1.348 (5)
C9—H9A	0.9300	N15—H15A	0.8600
C10—C11	1.375 (7)	N16—H16A	0.8600
C10—H10A	0.9300	N16—H16B	0.8600
C11—C12	1.371 (7)	N17—O1	1.233 (6)
C11—H11A	0.9300	N17—O2	1.249 (6)
C12—C13	1.375 (6)	N17—O3	1.250 (6)
C12—H12A	0.9300	N18—O4	1.252 (5)
C13—N5	1.347 (6)	N18—O5	1.256 (5)
C13—C14	1.452 (7)	N18—O6	1.258 (5)
C14—N6	1.273 (6)	N19—O9	1.229 (5)
C14—H14A	0.9300	N19—O8	1.238 (5)
C16—N8	1.329 (6)	N19—O7	1.249 (5)
C16—N7	1.354 (6)	N20—O12	1.236 (5)
C17—N9	1.328 (6)	N20—O11	1.252 (5)
C17—C18	1.387 (7)	N20—O10	1.281 (5)
C17—H17A	0.9300	O1W—H1WA	0.8500
C18—C19	1.359 (7)	O1W—H1WB	0.8501
C18—H18A	0.9300	O2W—H2WA	0.8499
C19—C20	1.394 (7)	O2W—H2WB	0.8500
C19—H19A	0.9300	O3W—H3WA	0.8502
C20—C21	1.383 (6)	O3W—H3WB	0.8500
C20—H20A	0.9300	O4W—H4WA	0.8500
C21—N9	1.347 (6)	O4W—H4WB	0.8502
			
N6—Zn1—N2	167.04 (16)	N10—C22—C21	116.6 (5)
N6—Zn1—N5	74.23 (16)	N10—C22—H22A	121.7
N2—Zn1—N5	97.52 (16)	C21—C22—H22A	121.7
N6—Zn1—N1	95.16 (16)	N12—C24—N11	116.2 (5)
N2—Zn1—N1	74.32 (16)	N12—C24—S3	121.4 (4)
N5—Zn1—N1	88.34 (15)	N11—C24—S3	122.4 (4)
N6—Zn1—S1	110.45 (11)	N13—C25—C26	123.0 (5)
N2—Zn1—S1	79.34 (12)	N13—C25—H25A	118.5
N5—Zn1—S1	91.99 (11)	C26—C25—H25A	118.5
N1—Zn1—S1	153.47 (13)	C27—C26—C25	119.1 (5)
N6—Zn1—S2	78.96 (12)	C27—C26—H26A	120.5
N2—Zn1—S2	108.58 (11)	C25—C26—H26A	120.5
N5—Zn1—S2	153.14 (12)	C26—C27—C28	118.9 (5)
N1—Zn1—S2	92.62 (11)	C26—C27—H27A	120.6
S1—Zn1—S2	98.79 (5)	C28—C27—H27A	120.6
N10—Zn2—N14	169.14 (15)	C29—C28—C27	118.6 (5)
N10—Zn2—N13	97.54 (15)	C29—C28—H28A	120.7
N14—Zn2—N13	73.92 (16)	C27—C28—H28A	120.7
N10—Zn2—N9	73.97 (15)	N13—C29—C28	122.7 (5)
N14—Zn2—N9	99.09 (15)	N13—C29—C30	115.1 (4)
N13—Zn2—N9	90.90 (15)	C28—C29—C30	122.1 (5)
N10—Zn2—S4	109.51 (11)	N14—C30—C29	116.6 (5)
N14—Zn2—S4	78.71 (11)	N14—C30—H30A	121.7
N13—Zn2—S4	152.61 (12)	C29—C30—H30A	121.7
N9—Zn2—S4	92.42 (11)	N16—C32—N15	116.1 (5)
N10—Zn2—S3	78.04 (11)	N16—C32—S4	121.2 (4)
N14—Zn2—S3	108.77 (11)	N15—C32—S4	122.7 (4)
N13—Zn2—S3	93.90 (11)	C1—N1—C5	118.7 (5)
N9—Zn2—S3	151.99 (12)	C1—N1—Zn1	127.7 (4)
S4—Zn2—S3	95.80 (5)	C5—N1—Zn1	113.5 (3)
C8—S1—Zn1	97.99 (18)	C6—N2—N3	121.0 (4)
C16—S2—Zn1	97.7 (2)	C6—N2—Zn1	118.6 (4)
C24—S3—Zn2	98.53 (19)	N3—N2—Zn1	120.4 (3)
C32—S4—Zn2	99.06 (19)	C8—N3—N2	119.7 (4)
N1—C1—C2	122.7 (6)	C8—N3—H3B	120.2
N1—C1—H1A	118.6	N2—N3—H3B	120.2
C2—C1—H1A	118.6	C8—N4—H4B	120.0
C3—C2—C1	119.0 (6)	C8—N4—H4C	120.0
C3—C2—H2A	120.5	H4B—N4—H4C	120.0
C1—C2—H2A	120.5	C9—N5—C13	118.6 (4)
C4—C3—C2	118.5 (5)	C9—N5—Zn1	126.8 (3)
C4—C3—H3A	120.7	C13—N5—Zn1	114.6 (3)
C2—C3—H3A	120.7	C14—N6—N7	120.2 (5)
C3—C4—C5	119.4 (6)	C14—N6—Zn1	118.6 (4)
C3—C4—H4A	120.3	N7—N6—Zn1	121.1 (3)
C5—C4—H4A	120.3	C16—N7—N6	118.8 (4)
N1—C5—C4	121.7 (5)	C16—N7—H7A	120.6
N1—C5—C6	116.2 (5)	N6—N7—H7A	120.6
C4—C5—C6	122.1 (5)	C16—N8—H8A	120.0
N2—C6—C5	117.2 (5)	C16—N8—H8B	120.0
N2—C6—H6A	121.4	H8A—N8—H8B	120.0
C5—C6—H6A	121.4	C17—N9—C21	118.1 (4)
N4—C8—N3	115.3 (5)	C17—N9—Zn2	127.2 (3)
N4—C8—S1	122.1 (4)	C21—N9—Zn2	114.7 (3)
N3—C8—S1	122.6 (4)	C22—N10—N11	120.9 (4)
N5—C9—C10	121.5 (5)	C22—N10—Zn2	117.9 (4)
N5—C9—H9A	119.3	N11—N10—Zn2	121.1 (3)
C10—C9—H9A	119.3	C24—N11—N10	118.5 (4)
C11—C10—C9	119.1 (5)	C24—N11—H11B	120.7
C11—C10—H10A	120.5	N10—N11—H11B	120.7
C9—C10—H10A	120.5	C24—N12—H12B	120.0
C12—C11—C10	119.3 (5)	C24—N12—H12C	120.0
C12—C11—H11A	120.4	H12B—N12—H12C	120.0
C10—C11—H11A	120.4	C25—N13—C29	117.7 (4)
C11—C12—C13	118.5 (5)	C25—N13—Zn2	126.7 (3)
C11—C12—H12A	120.7	C29—N13—Zn2	115.6 (3)
C13—C12—H12A	120.7	C30—N14—N15	120.2 (4)
N5—C13—C12	123.0 (5)	C30—N14—Zn2	118.6 (4)
N5—C13—C14	115.3 (4)	N15—N14—Zn2	121.1 (3)
C12—C13—C14	121.7 (5)	N14—N15—C32	118.4 (4)
N6—C14—C13	117.2 (5)	N14—N15—H15A	120.8
N6—C14—H14A	121.4	C32—N15—H15A	120.8
C13—C14—H14A	121.4	C32—N16—H16A	120.0
N8—C16—N7	115.1 (5)	C32—N16—H16B	120.0
N8—C16—S2	121.5 (4)	H16A—N16—H16B	120.0
N7—C16—S2	123.3 (4)	O1—N17—O2	120.8 (5)
N9—C17—C18	123.4 (5)	O1—N17—O3	120.7 (5)
N9—C17—H17A	118.3	O2—N17—O3	118.5 (6)
C18—C17—H17A	118.3	O4—N18—O5	120.5 (4)
C19—C18—C17	118.2 (5)	O4—N18—O6	120.0 (5)
C19—C18—H18A	120.9	O5—N18—O6	119.5 (5)
C17—C18—H18A	120.9	O9—N19—O8	120.5 (5)
C18—C19—C20	119.8 (5)	O9—N19—O7	120.2 (6)
C18—C19—H19A	120.1	O8—N19—O7	119.3 (5)
C20—C19—H19A	120.1	O12—N20—O11	121.5 (5)
C21—C20—C19	118.3 (5)	O12—N20—O10	120.1 (5)
C21—C20—H20A	120.9	O11—N20—O10	118.4 (5)
C19—C20—H20A	120.9	H1WA—O1W—H1WB	112.2
N9—C21—C20	122.1 (5)	H2WA—O2W—H2WB	127.8
N9—C21—C22	115.9 (4)	H3WA—O3W—H3WB	100.8
C20—C21—C22	121.9 (5)	H4WA—O4W—H4WB	108.9

### Hydrogen-bond geometry (Å, °)

**Table:**

*D*—H···*A*	*D*—H	H···*A*	*D*···*A*	*D*—H···*A*
N3—H3B···O7	0.86	2.19	2.966 (6)	150
N4—H4B···O7	0.86	2.17	2.957 (6)	153
N4—H4C···O3W	0.86	2.03	2.840 (5)	157
N7—H7A···O1	0.86	1.96	2.813 (6)	172
N8—H8A···O2	0.86	2.08	2.906 (6)	160
N8—H8B···O6^i^	0.86	2.04	2.870 (6)	163
N11—H11B···O10	0.86	1.87	2.710 (5)	167
N12—H12B···O11	0.86	2.02	2.876 (6)	173
N12—H12C···O8^ii^	0.86	2.27	3.114 (6)	168
N15—H15A···O4W^iii^	0.86	1.87	2.733 (5)	178
N16—H16A···O6^iii^	0.86	2.11	2.909 (6)	155
N16—H16B···O4^iv^	0.86	2.02	2.873 (5)	173
O1W—H1WA···O4^v^	0.85	2.32	3.042 (5)	144
O1W—H1WB···O3	0.85	2.39	3.041 (6)	134
O2W—H2WA···O5^iv^	0.85	2.33	2.927 (6)	128
O2W—H2WB···O3	0.85	2.47	3.141 (6)	136
O3W—H3WA···O10^vi^	0.85	2.22	2.800 (5)	126
O3W—H3WB···O9^vii^	0.85	2.24	2.978 (6)	145
O4W—H4WA···O2W	0.85	2.09	2.746 (6)	133
O4W—H4WB···O6	0.85	2.55	2.958 (5)	111

Symmetry codes: (i) −*x*+1, −*y*+1, −*z*+1; (ii) *x*, −*y*+1/2, *z*+1/2; (iii) *x*, −*y*+3/2, *z*+1/2; (iv) −*x*+1, *y*−1/2, −*z*+3/2; (v) *x*, −*y*+3/2, *z*−1/2; (vi) −*x*, *y*−1/2, −*z*+1/2; (vii) −*x*, *y*+1/2, −*z*+1/2.
